# Genotyping of polymorphic effectors of *Toxoplasma gondii* isolates from China

**DOI:** 10.1186/s13071-017-2527-4

**Published:** 2017-11-21

**Authors:** Weisheng Cheng, Cong Wang, Ting Xu, Fang Liu, Faustina Pappoe, Qingli Luo, Yuanhong Xu, Fangli Lu, Jilong Shen

**Affiliations:** 1 Department of Microbiology and Parasitology, Anhui Provincial Laboratory of Parasitology and the Key Laboratory of Zoonoses, School of Basic Medicine, Anhui Medical University, Hefei, 230022 China; 2 Department of Parasitology of Zhongshan School of Medicine, Sun Yat-sen University, Guangzhou, 510080 China; 3 Department of Clinical Laboratory, the First Affiliated Hospital of Anhui Medical University, Hefei, 230022 China; 4 Department of Microbiology and Immunology, School of Medical Sciences, College of Health and Allied Sciences, University of Cape Coast, Cape Coast, Ghana

**Keywords:** *Toxoplasma gondii*, Virulence effectors, PCR-RFLP, Bioinformatics analyses

## Abstract

**Background:**

*Toxoplasma gondii* is an opportunistic protozoan apicomplexan and obligate intracellular parasite that infects a wide range of animals and humans. Rhoptry proteins 5 (ROP5), ROP16, ROP18 and dense granules 15 (GRA15) are the important effectors secreted by *T. gondii* which link to the strain virulence for mice and modulate the host’s response to the parasite. Little has been known about these molecules as well as GRA3 in type Chinese 1 strains that show polymorphism among strains of archetypical genotypes. This study examined the genetic diversity of these effectors and its correlated virulence in mice among *T. gondii* isolates from China.

**Results:**

Twenty-one isolates from stray cats were detected, of which 15 belong to Chinese 1, and 6 to ToxoDB #205. Wh6 isolate, a Chinese 1 strain, has an avirulent phenotype. PCR-RFLP results of ROP5 and ROP18 presented few variations among the strains. Genotyping of GRA15 and ROP16 revealed that all the strains belong to type II allele except Xz7 which carries type I allele. ROP16 amino acid alignment at 503 locus demonstrated that 17 isolates are featured as type I or type III (ROP16_I/III_), and the other 4 as type II (ROP16_II_). The strains investigated may be divided into four groups based on GRA3 amino acid alignment, and all isolates of type Chinese 1 belong to the μ-1 allele except Wh6 which is identical to type II strain.

**Conclusions:**

PCR-RFLP and sequence alignment analyses of ROP5, ROP16, ROP18, GRA3, and GRA15 in *T. gondii* revealed that strains with the same genotype may have variations in some of their key genes. GRA3 variation exhibited by Wh6 strain may be associated with the difference in phenotype and pathogenesis.

**Electronic supplementary material:**

The online version of this article (10.1186/s13071-017-2527-4) contains supplementary material, which is available to authorized users.

## Background

*Toxoplasma gondii* is an obligate intracellular protozoon that can infect a broad spectrum of vertebrate hosts including humans. The infection with *T. gondii* in domestic animals causes abortion and leads to great economic losses in livestock production. In humans, *Toxoplasma* infection usually does not lead to obvious clinical symptoms and signs [1]. Latent *Toxoplasma* infection, however, may become activated in immunocompromised individuals, causing severe or life-threatening disseminated toxoplasmosis such as encephalitis or lethargy [2]. Recent reports have shown that 3 to 97% of HIV patients have concurrent recessive *Toxoplasma* infection [3] and more than one-third of encephalitis in AIDS patients are caused by *Toxoplasma* infection [4]. Long-term chemotherapy of tumor patients, as well as autoimmune disease of patients with continuous glucocorticoid administration, may result in activation of the recessive infection and risk of toxoplasmosis [5]. Additionally, some mental disorders such as schizophrenia, suicide, or even traffic and workplace accidents are believed to be associated with chronic *Toxoplasma* infection [6].

In the biological taxonomy, the genus *Toxoplasma* contains only one species, *Toxoplasma gondii*. However, genotyping results of the strains collected from human and animals around the world show a rich genetic diversity [7] which points to the fact that the distribution of *T. gondii* genotypes varies greatly with geographical locations [8]. So far more than 200 genotypes have been recorded in the *T. gondii* database (http://toxodb.org/toxo). In North America and Europe, *T. gondii* has three archetypal clonal lineages known as types I, II and III which exhibit remarkable phenotypic differences [9]. Comparatively, isolates in Central and South America show an extremely complex genetic structure. In China, genotype Chinese 1 (*Toxo*DB#9) dominates in the ten types identified [10–19], which is quite different from those of the clonal lineages in the other continents of the world.

*Toxoplasma gondii* has evolved a number of strategies to subvert its host’s immune responses. Previous studies indicated that ROP16I/III carried in types I and III strains induces alternatively activated macrophage (M2) in host innate immunity to *Toxoplasma* infection, modulating host signaling pathways and producing virulence in mice [20]. Alvarez et al. [21] analyzed the nucleotide sequence of ROP16 and found that 83.3% (10/12) of the isolates from patients with ocular retinochoroiditis caused by toxoplasmosis had mouse virulence associated with ROP16_I_, while all strains (100%) isolated from meat were avirulent for mice. Interestingly, we found that all the Chinese 1 isolates, including virulent Wh3 and avirulent Wh6 strains, shared the genes of GRA15_II_ with type II strains and ROP16_I/III_ with types I or III strains [22]. We observed that *rop16*_*I/III*_ deficient Wh3 strain (data unpublished) did not result in a remarkable attenuation of virulence in mice, suggesting that ROP16_I/III_ in the parasite with GRA15_II_ background is not closely associated in the virulence of Chinese 1 strains.

Previous genetic mapping of crosses between clonal type I, II, and III strains of *T. gondii* identified the rhoptry kinase ROP18 and rhoptry pseudokinase ROP5 as virulence factors of the three archetypal clonal lineages. They function together to block innate immune mechanisms activated by IFN-γ in murine hosts [23–26]. However, the structure of ROP18 and ROP5 as well as other virulence factors in genetically variative Chinese 1 strains remains unknown. Dense granule protein 3 (GRA3) is known to be associated with the parasitophorous vacuole membrane (PVM) [27] and plays a role in acute infection phase of type II strains [28]. Our previous study revealed that GRA3 has a dramatically high expression in the avirulent Wh6 strain when compared with the virulent Wh3 strain of type Chinese 1 [10], suggesting its involvement in strain virulence in mice.

Consequently, we analyzed the polymorphism of rhoptry proteins of ROP16, ROP18, ROP5 and dense granule proteins of GRA15 and GRA3 of type Chinese 1 strains to (i) explore the characteristics of the effectors that are associated with host immunity subversion and mouse virulence among the strains and (ii) to reveal the polymorphism-related pathogenesis of *T. gondii* in mice.

## Methods

### Mice

Female Swiss Webster (SW) mice (specific pathogen free) aged 6 to 8 weeks were obtained from the Biomedical Research Institute of Nanjing University, China. The mice were treated in compliance with the Care and Use of Laboratory Animals of the National Institutes of Health. Ethical permission was obtained from the Institutional Review Board of the Institute of Biomedicine at Anhui Medical University (Permit No. AMU26–081108).

### *Toxoplasma gondii* DNA

Genomic DNA of the *T. gondii* strains were extracted from the ascitic fluid of mice infected with each isolate using a QIAamp® DNA Mini kit (Qiagen, Hilden, Germany) following the manufacturer’s protocols. Genomic DNA stocks were stored at -80 °C until analyzed.

### PCR-RFLP protocols

GRA15, ROP16 and ROP18 PCR-RFLP protocols were followed in accordance with a previous study [29]. ROP5 allele was analyzed as previously described [29, 30]. For each locus, PCR was run on genomic DNA using external primers, followed by PCR amplification with internal primers. All sequences of the primers for each locus are shown in Table 1. A Phusion High-Fidelity PCR Kit (Thermo Fisher Scientific,California, USA) was used for PCR amplification with individual marker primers and a thermocycler program of 5 min at 94 °C, 37 cycles of 30 s at 94 °C, 30 s at 58 °C, and 45 s at 72 °C, and finally, 5 min at 72 °C. PCR products were purified after agarose gel electrophoresis conformation using Axygen PCR clean up kit (Axygen,California, USA). The new primers used in this study are shown in Table 1.

**Table 1:** PCR primes for genotyping ROP16, GRA3 and GRA15

Marker	External primers for multiplex PCR	Internal primers for nested PCR	Restriction digestion
ROP16	ROP16-Fext: TCGTCCCGAATGCTGATGCCACGTC	ROP16-Fint: AAGCAACCGTGGTACGTCGAGGTTC	No restriction enzyme needed. Sequencing using double deoxidizing terminal cessation method
	ROP16-Rext: ATGCCCAAAGCCGTGGACATCGATC	ROP16-Rint: TCCATGCGCGAATCCAAGTTCGTG	
GRA15	GRA15-Fext2: GCGTACATGGTTATGCGACG	GRA15-Fint2: GGTCATTGTCTGCAGACTGAT	No restriction enzyme needed. Sequencing using double deoxidizing terminal cessation method
	GRA15-Rext2: CATTCCAGTCCCTAAGTTCCCT	GRA15-Rint1: CCCTTATCGGTTTTTGGTCA	
GRA3	GRA3-Fext: CCTTATTTAATGTTAGATCATCCCG	GRA3-Fint: AGGTACGCGTCGAGTAACCAGT	No restriction enzyme needed. Sequencing using double deoxidizing terminal cessation method
	GRA3-Rext: ACACCCGGTAGCAAGCGTTCA	GRA3-Rint: CCCGAGAGAGACTGGCACGA	

### Amplification of ROP16, GRA3 and GRA15 loci

ROP16 nucleotides 1424–1614 [21] and the full length of nucleotides of GRA3 and GRA15 were amplified using a nested PCR amplification strategy. Primers (Table 1) were synthesized by Sangon Biotech, Shanghai, China. The PCR amplification program was as follows: 5 min of predenaturation at 94 °C followed by 39 cycles, each containing 30 s of denaturation at 94 °C, 30 s at the optimum annealing temperature, and 30 s or 2 min for extension at 72 °C, and finally 10 min of additional extension at 72 °C. All PCRs were performed in a T-Gradient Thermoblock (Biometra, Goettingen, Germany). PCR products were purified after agarose gel electrophoresis conformation and sequenced by Sangon Biotech using a 3730XL DNA sequencer. Sequence alignment and phylogenetic trees were derived using CLC Genomics Workbench 7.7 (Qiagen).

### Fatality assay

Virulence of *T. gondii* strains was tested based on the previous mouse bioassay data. The virulence difference between Wh3 and Wh6 strains has been noted in long-term serial mouse passages. Here mice were infected with 1000 tachyzoites by intraperitoneal injection and observed for 30 days post-infection. *Toxoplasma gondii* strains that caused 0–29%, 30–79% and 80–100% mortality of mice were considered non-virulent, intermediate and virulent, respectively [30]. The previous data on mouse mortality were used in the present study instead of repeating dose-dependent virulent tests in order to preserve animals.

## Results

### Fatality assay of type Chinese 1 and ToxoDB #205 strains

We assessed the mouse mortality of type Chinese 1 and ToxoDB #205 strains in combination with the data of previous studies. Mice were intraperitoneally infected with 1000 tachyzoites and mouse survival was observed for 30 days post-infection. The results showed that all of these strains presented a virulent phenotype in mice except for the Wh6 isolate which has the only avirulent phenotype [10].

### Genotyping of *T. gondii* isolates at ROP18, ROP5, ROP16 and GRA15 loci by PCR-RFLP method

We genotyped the ROP18, ROP5, ROP16 and GRA15 loci of 21 isolates collected from animals and compared the results with the reference strains. For ROP18, none of the isolates contained the upstream insertion sequence. All the Chinese 1 and ToxoDB #205 strains belong to *rop18* allele 2 except for Xz7 strain which carries *rop18* allele 1 (Table 2).

**Table 2:** PCR-RFLP genotyping of *T. gondii* isolates with polymorphic loci of ROP5, ROP16, ROP18 and GRA15

Strain ID	ToxoDB genotype #	ROP5	ROP18	GRA15^a^	ROP16	Virulence	Mouse mortality (%)	Reference
GT1	10	1	I	I/III	I	Vir	100	[40]
Me49	1	2	II	II	II	Int	40	[40]
VEG	2	3	III	I/III	III	Non	13	[40]
Wh2	9	5	II	II	II	Vir	100	[13]
Wh3	9	5	II	II	II	Vir	100	[13]
Wh4	9	5	II	II	II	Vir	80	[13]
Wh5	9	5	II	II	II	Vir	100	[13]
Wh6	9	5	II	II	II	Non	45	[9]
Wh7	9	5	II	II	II	Vir	100	[13]
Wh10	9	5	II	II	II	Vir	100	This study
Wh12	9	5	II	II	II	Vir	100	[12]
Wh13	9	5	II	II	II	Vir	100	This study
Wh14	9	5	II	II	II	Vir	100	[12]
Xz34	9	5	II	II	II	Vir	100	This study
Xz38	9	5	II	II	II	Vir	100	This study
Xz7	205	7	I	I/III	I	Vir	100	This study
Xz8	205	5	II	II	II	Vir	100	[12]
Xz9	205	7	II	II	II	Vir	100	This study
Xz37	205	7	II	II	II	Vir	100	This study
Xz39	205	7	II	II	II	Vir	100	This study
Xz40	205	7	II	II	II	Vir	100	This study
Ys1	9	5	II	II	II	Vir	80	[13]
Ys2	9	5	II	II	II	Vir	100	[13]
Gy5	9	5	II	II	II	Vir	100	[11]

*Abbreviations*: Int, intermediate virulence; Non, avirulence; Vir, virulence

^a^ROP16II: examined by PCR-RFLP genotyping, indicating the restricted fragment of ROP16 is identical to that of type II strains

Additionally, we sequenced ROP18 of the Wh3 strain and its amino acid alignment showed a 96 or 99% positivity compared with ROP18 of virulent GT1 or avirulent ME49 strains (data not shown).

ROP5 is the major determinant of acute virulence in mice and was found to have allele 5 in all Chinese 1 strains but allele 7 in ToxoDB#205 and allele 5 in Xz8 strains (Table 2). The *rop5* allele 7 was found to be a unique genotype which could not be digested by *Fsp*BI.

ROP16_I/III_ of type I and type III strains is known to directly induce STAT3/STAT6 phosphorylation and evoke M2 dominant innate immune response to the parasite. All the Chinese 1 and ToxoDB #205 strains have ROP16 allele 2 except for the Xz7 strain which carries ROP16 allele 1.

Compared with type I and type III strains, GRA15_II_ of type II strain features with an 84-amino acid deficiency, coinciding with the deletion of nucleotides at the genome level. All of the isolates carry GRA15_II_ except for ToxoDB #205 Xz7 strain which has GRA15_I/III_ (Table 2). We also analyzed the full length of GRA15 and the results showed that all the Chinese 1 and ToxoDB #205 strains, but not Xz7 strain, are identical to type II strain (Additional file 1: Figure S1).

### Amino acid residue of ROP16 at position 503

**Fig. 1:**
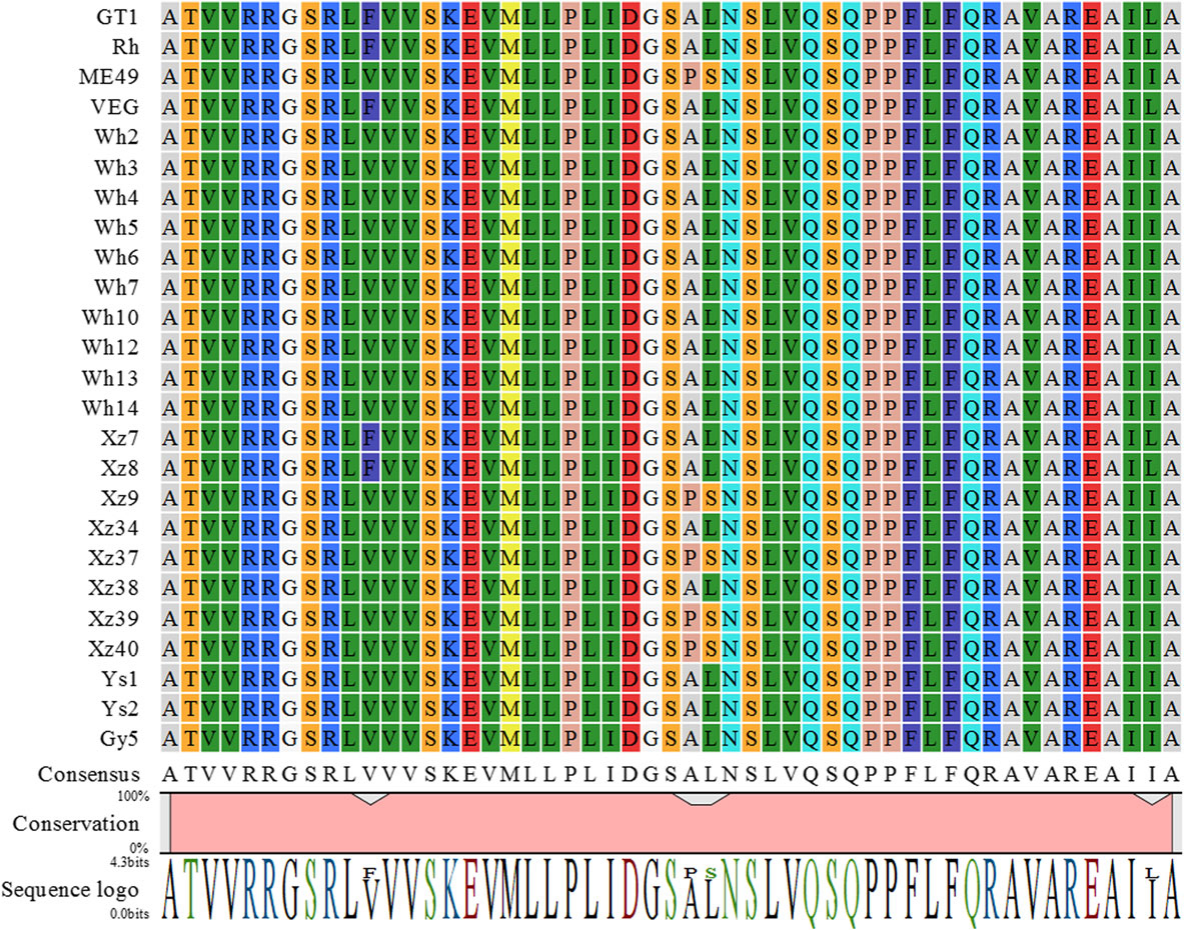
ROP16 translations alignment. Amino acid alignment shows that at position 503 (arrow) all strains of Chinese 1 and strains of Xz7 and Xz8 of ToxoDB#205 are characterized by carrying leucine, at positions 486 in type Chinese 1 and ToxoDB 205# strains were identical to those of archetypal type II strains except for Xz7 and Xz8 trains which were identical to archetypal type I strain

We specifically analyzed the predicted amino acid sequence (based on DNA sequence) of ROP16 and found that all strains of Chinese 1 and strains of Xz7 and Xz8 of ToxoDB #205 are characterized by carrying leucine at position 503 while Xz9, Xz37, Xz39 and Xz40 strains of ToxoDB#205 presented serine. Additionally, amino acid of ROP16 at position 486 in Chinese 1 and ToxoDB#205 strains are homologous to that of archetypal type II strains except for Xz7 and Xz8 strains that are identical to archetypal type I strain, as shown in Table 3 and Fig. 1.

**Table 3:** Genotype ROP16 according to amino acid residue of position 503 and full-length alignment genotyping of GRA3

Strain ID	ToxoDB genotype#	ROP16^a^	GRA3	Virulence	References
GT1	10	I/III	I	Vir	[40]
Me49	1	II	II	Int	[40]
VEG	2	I/III	III	Non	[40]
Wh2	9	I/III	μ-1	Vir	[13]
Wh3	9	I/III	μ-1	Vir	[13]
Wh4	9	I/III	μ-1	Int	[13]
Wh5	9	I/III	μ-1	Vir	[13]
Wh6	9	I/III	II	Non	[10]
Wh7	9	I/III	μ-1	Vir	[13]
Wh10	9	I/III	μ-1	Vir	This study
Wh12	9	I/III	μ-1	Vir	[12]
Wh13	9	I/III	μ-1	Vir	This study
Wh14	9	I/III	μ-1	Vir	[12]
Xz34	9	I/III	μ-1	Vir	This study
Xz38	9	I/III	μ-1	Vir	This study
Xz7	205	I/III	I	Vir	This study
Xz8	205	I/III	II	Vir	[12]
Xz9	205	II	μ-2	Vir	This study
Xz37	205	II	μ-2	Vir	This study
Xz39	205	II	μ-2	Vir	This study
Xz40	205	II	μ-2	Vir	This study
Gy5	9	I/III	μ-1	Vir	[11]

*Abbreviations*: Int, intermediate virulence: Non, avirulence; Vir, virulence

^a^ROP16_I/III_: identified by sequencing, indicating the amino acid residue of ROP16 at position 503 is leucine (seen in type I or type III strain) instead of serine (seen in type II strain). ROP16_I/III_ is widely used since it more precise

### Full length alignment of GRA3

**Fig. 2:**
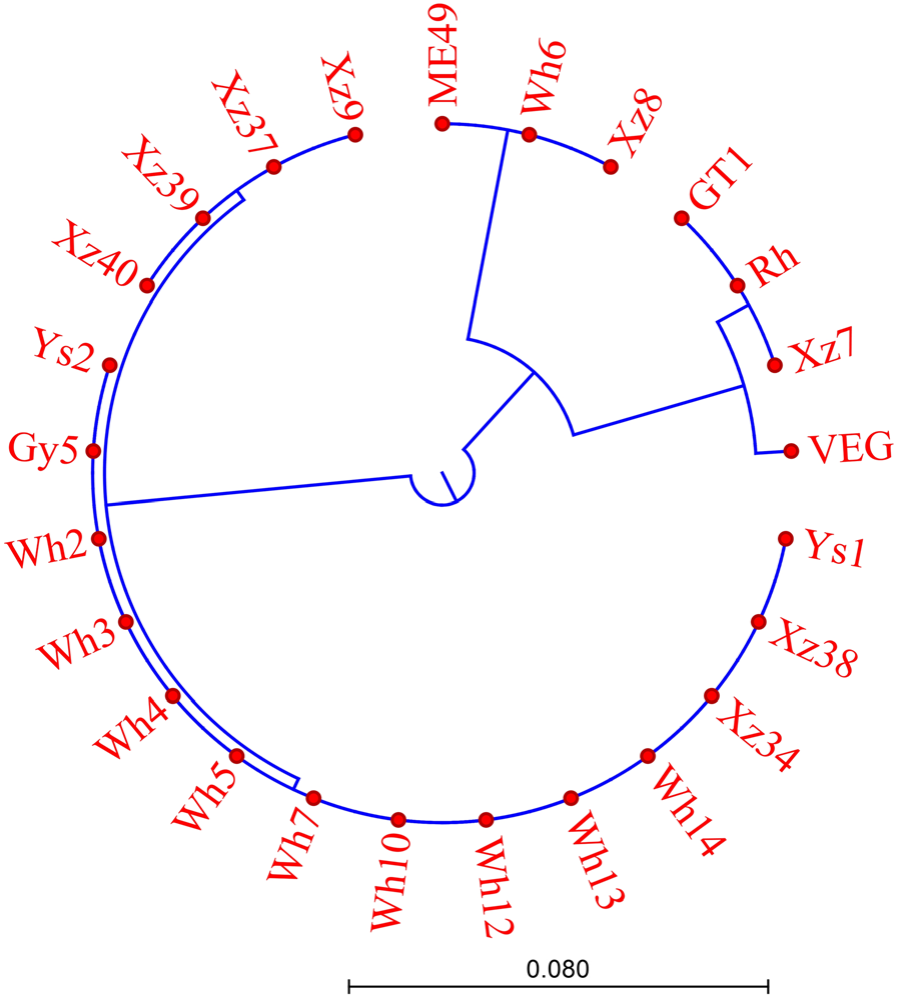
Phylogenetic tree of GRA3 translation inferred by using the neighbor-joining method based on the Kimura protein model. Analyses were conducted in CLC Genomics Workbench 7.7

The *gra3* full length of nucleotides was amplified in the strains investigated. The coding sequences were aligned, followed by construction of the phylogenetic tree. We found that the sequences could be categorized into 5 types, the archetypal types I, II and III and μ-1 and μ-2 (shown in Fig. 2, Table 3 and Additional file 2: Figure S2). The Xz7 was identical to GT1 and RH strains of type I, while type III GRA3 had a slight change at the 166–168 amino acid positions when compared with type I. Also, the C to T change at position 661 of the open reading frame leads to early appearance of termination which in turn results in a 2 amino acids deficiency of type II GRA3 compared with types I and III.

All strains contained the same length of GRA3 as type II strain except the Xz7 strain. GRA3 of Wh6 and Xz8 strains were homologous to that of type II. GRA3 of strains Wh2, Wh3, Wh4, Wh5, Wh7, Wh10, Wh12, Wh13, Wh14, Xz34, Xz38, Gy5, Ys1 and Ys2 belonged to the μ-1 allele whereas that of Xz9, Xz37, Xz39, and Xz40 belong to the μ-2 allele. Only one amino acid variation at position 151 was noted in μ-1 and μ-2 alleles. The amino acid sequence from 144 to 180 of μ-1 and μ-2 alleles was found to have the mutation cluster region constituting a major part of all mutations.

## Discussion

*Toxoplasma gondii* is known to have the ability to subvert any sort of nucleated cells behavior and is arguably the most successful protozoan. Recent investigations indicated that type Chinese 1 dominates in China, which is genetically and phenotypically different from those in other continents of the world [31]. *Toxoplasma gondii* has three unique organelles: microneme, rhoptry, and granule. These organelles secrete effector molecules into host cytosol to modulate host signaling pathways and influence parasite development in the host. Previous studies have shown that some effectors have polymorphism among strains or genotypes, leading to various pathogenic mechanisms [24]. Analyzing the sequence variation of the effectors in pathogenicity or virulence-associated factors is crucial for understanding and evaluating the potential pathogenesis of *T. gondi.*

Gene allele types of isolates from China showed a slight difference from the reference strains but still presented generality among type Chinese 1 strains. RFLP genotyping of those factors uncovered the consistency in Chinese 1 strains. In ToxoDB#205 strains, GRA15 and ROP18 of Xz7 and ROP5 of Xz8 showed variations compared with other strains. However, comparison of ROP18 sequencing of the Chinese 1 Wh3 strain with the data online showed a homology of 98–99% identity to other strains with different virulence in the other continents (TgGZ2, GZ3, GZ7, GZ8, DEG, ME49, TgPgPYS, QHO, GT1, TgCatBr18, PTG, and RH, etc.). Amino acid alignments revealed 96% or 99% positivities of ROP18 of the Chinese 1 Wh3 strain in comparison with that of GT1 or ME49, respectively, suggesting that sequence variations of ROP18 indeed exists but was not able to be identified by the PCR-RFLP analysis in the present study.

Shwab et al. [30] analyzed four rhoptry protein gene loci including ROP18, ROP5, ROP16 and ROP17 of 240 globally distributed *T. gondii* strains. The results revealed that the ROP18 and ROP5 gene allele types could be used to predict strain virulence in mice. ROP18 has 4 alleles; alleles 1 and 4 show association with a virulent phenotype, while allele 2 and 3 strains show a low virulence in mice [30]. However, ROP5 carries 6 alleles and alleles 1, 3, 4 and 6 are involved in high mouse virulence [30]. Strains containing ROP18 allele 2 and ROP5 allele 5 tend to have an avirulent phenotype in mice. In the present study, strains containing both ROP18_2_ and ROP5_5_ show a virulent phenotype in mice except the Wh6 strain. This result is inconsistent with the previous report using RFLP [30], suggesting that genotyping of ROP5 and ROP18 using the current RFLP strategy is inadequate to predict the virulence of Chinese 1 isolates. The virulence differences still might be attributable to undescribed variation at these loci or to the interaction of ROP5_2_ with ROP18_2_ and ROP17 [32], and/or to other molecules that remain unidentified.

GRA15_II_ in type II strains mediates NF-kB nuclear translocation, drives macrophages towards a classically activated phenotype (M1) and promotes host innate immunity against *Toxoplasma* infection [33]. Meanwhile, ROP16_I/III_ of types I and III has the ability to phosphorylate both STAT3 and STAT6 which results in the alternative activation of macrophages (M2), facilitating replication of parasites within the host cells [20, 34, 35]. In order to confirm the allele of ROP16 and GRA15, we analyzed the polymorphism of GRA15 and ROP16 through PCR-RFLP, and the results showed that all of the isolates contained the allele 2 GRA15 and ROP16 except for the Xz7 strain.

Yamamoto et al. [36] have shown that the amino acid residue of ROP16_II_ at position 503 in type II strains (e.g. PRU, ME49) is serine (ROP16 S503) which has no phosphorylation activity of STAT3/6 kinase. The types I and III strains (RH, CTG), however, are featured with ROP16 L503 (ROP16_I/III_) instead of S503 (ROP16_II_). ROP16_I/III_ is able to phosphorylate STAT3/6, drive macrophage to M2 polarization and help the parasite with its multiplication in macrophages [36]. This may result in systemic infection and even kill the host. Thus, the strain-associated polymorphism of GRA15 and ROP16 determines the fate of the parasite and the outcome of *Toxoplasma* infection. Interestingly, in the present study, we found that the amino acid residue of ROP16 at position 503 is leucine (i.e. ROP16 L503, ROP16_I/III_) in all of the isolates of type Chinese 1 and some isolates (Xz7, Xz8) of ToxoDB#205 identified in China mainland. Additionally, we noted that all of the strains of type Chinese 1 and some of ToxoDB#205, regardless of their mouse virulence, carry GRA15_II,_ which is identical to GRA15_II_ in type II strains [35]. The characteristic of Chinese 1 strains of *T. gondii* that have both key effectors of ROP16_I/III_ and GRA15_II_ implicates the unique mechanism of the host immune response and pathogenesis in Chinese 1 *T. gondii* infection.

Dense granule protein 3 (GRA3) is known to be secreted by the parasites after invasion and appears at the parasitophorous vacuole membrane (PVM) [27]. It plays a role in acute infection of type II strains [28] and reduces growth rate under starving conditions in culture and contributes to virulence in mice [28, 37]. Our previous investigations revealed that the GRA3 expression level in Wh6, the avirulent strain of Chinese 1, dramatically increased when compared with virulent Wh3 and RH strains [10, 22]. We aligned the amino acid sequence of GRA3 and found that among Chinese 1 strains, only Wh6 shares the GRA3 homologue sequence with the type II strain. The precise function of GRA3 and its polymorphism remains unclear although it is believed to interact with host cell calcium modulating ligand (CAML) [38].

Furthermore, a more comprehensive analysis of type Chinese 1 strains is needed at the genomic DNA and transcriptome level. The development and application of CRISPR-Cas9 technology will enable us to precisely manipulate target genes to extend the power of reverse genetics in *Toxoplasma* [39].

## Conclusions

The present study examined sequence variations of ROP5, ROP16, ROP18, GRA3 and GRA15 genes in 21 Chinese 1 and ToxoDB#205 isolates of *T. gondii* from China. We demonstrated that the majority of those isolates are featured with the phenotypic ROP16_I/III_ of type I and type III strains and GRA15_II_ of type II strains. This strongly suggests that the characterized polymorphism of the crucial effectors of ROP16 and GRA15 in Chinese 1 strains may result in a significantly different outcome of *Toxoplasma* infection through the subversion of the host’s innate immunity. Furthermore, the Wh6 strain contains type II GRA3 which is unique in Chinese 1 strains and may lead to phenotype differences. Additionally, the present results indicate that genotyping of the loci including ROP5 and ROP18 with a PCR-RFLP strategy that is globally used may be inadequate to predict the virulence of Chinese 1 isolates of *T. gondii.*

## Additional files

Additional file 1: Figure S1. GRA15 translations alignment**.** Analyzing the full length of GRA15, the result shows that all Chinese 1 and ToxoDB #205 strains (but not Xz7 strain) were identical to the type II strain, which has an 84 aa deletion from position 519 to 602. (TIFF 3957 kb)

Additional file 2: GRA3 translations alignment. Aligning the full length of GRA3 translations of those strains, shows that Xz7 is the only one which has a longer length compared with the others. GRA3 of Wh6 and Xz8 strains are homologous to that of type II. The amino acid sequence from 131 to 180 is the mutation cluster region constituting a major part of all mutations. (TIFF 4457 kb)

## Abbreviations

CAML: calcium modulating ligand
GRA15: dense granules 15
GRA3: granule protein 3
M1: classically activated phenotype
M2: alternatively activated macrophages
PVM: parasitophorous vacuole membrane
ROP5: Rhoptry proteins 5
SW: Swiss Webster

## References

[1] Dubey JP. Toxoplasmosis of Animals and Humans. 2nd ed. Boca Raton: Taylor and Francis; 2010.

[2] Schmidt M, Sonneville R, Schnell D, Bige N, Hamidfar R, Mongardon N (2013). Clinical features and outcomes in patients with disseminated toxoplasmosis admitted to intensive care: a multicenter study. Clin Infect Dis.

[3] Nissapatorn V (2009). Toxoplasmosis in Hiv/Aids: a living legacy. Southeast Asian J Trop Med Public Health.

[4] Luma HN, Tchaleu BC, Temfack E, Doualla MS, Ndenga DP, Mapoure YN, et al. HIV-associated central nervous system disease in patients admitted at the Douala General Hospital between 2004 and 2009: a retrospective study. AIDS Res Treat. 2013;2013:709810. 10.1155/2013/709810 PMC3600338 23533732

[5] Wang L, He LY, Meng DD, Chen ZW, Wen H, Fang GS, et al. Seroprevalence and genetic characterization of *Toxoplasma gondii* in cancer patients in Anhui Province, eastern China. Parasit Vectors. 2015;8:162. 10.1186/s13071-015-0778-5 PMC4379604 25889184

[6] Wang T, Tang ZH, Li JF, Li XN, Wang X, Zhao ZJA (2013). Potential association between *Toxoplasma gondii* infection and schizophrenia in mouse models. Exp Parasitol.

[7] Su C, Khan A, Zhou P, Majumdar D, Ajzenberg D, Darde ML, et al. Globally diverse *Toxoplasma gondii* isolates comprise six major clades originating from a small number of distinct ancestral lineages. Proc Natl Acad Sci USA. 2012;109(15):5844–9. 10.1073/pnas.1203190109 PMC3326454 22431627

[8] Lehmann T, Marcet PL, Graham DH, Dahl ER, Dubey JP. Globalization and the population structure of *Toxoplasma gondii*. Proc Natl Acad Sci USA. 2006;103(30):11423–8. 10.1073/pnas.0601438103 PMC1544101 16849431

[9] Saeij JPJ, Boyle JP, Boothroyd JC (2005). Differences among the three major strains of *Toxoplasma gondii* and their specific interactions with the infected host. Trends Parasitol.

[10] Li M, Mo XW, Wang L, Chen H, Luo QL, Wen HQ (2014). Phylogeny and virulence divergency analyses of *Toxoplasma gondii* isolates from China. Parasit Vectors.

[11] Wang L, Cheng HW, Huang KQ, YH X, Li YN, Du J, et al. *Toxoplasma gondii* prevalence in food animals and rodents in different regions of China: isolation, genotyping and mouse pathogenicity. Parasit Vectors. 2013;6:273. 10.1186/1756-3305-6-273 PMC3849108 24330536

[12] Wang L, Chen H, Liu DH, Huo XX, Gao JM, Song XR (2013). Genotypes and mouse virulence of *Toxoplasma gondii* isolates from animals and humans in China. PLoS One.

[13] Chen ZW, Gao JM, Huo XX, Wang L, Yu L, Halm-Lai F (2011). Genotyping of *Toxoplasma gondii* isolates from cats in different geographic regions of China. Vet Parasitol.

[14] Dubey JP, Zhu XQ, Sundar N, Zhang H, Kwok OC, Genetic SC (2007). Biologic characterization of *Toxoplasma gondii* isolates of cats from China. Vet Parasitol.

[15] Wang H, Wang T, Luo Q, Huo X, Wang L, Liu T (2012). Prevalence and genotypes of *Toxoplasma gondii* in pork from retail meat stores in eastern China. Int J Food Microbiol.

[16] Zhou P, Zhang H, Lin RQ, Zhang DL, Song HQ, Su C (2009). Genetic characterization of *Toxoplasma gondii* isolates from China. Parasitol Int.

[17] Zhou Y, Zhang H, Cao J, Gong H, Zhou J (2013). Isolation and genotyping of *Toxoplasma gondii* from domestic rabbits in China to reveal the prevalence of type III strains. Vet Parasitol.

[18] Tian YM, Huang SY, Miao Q, Jiang HH, Yang JF, Su C (2014). Genetic characterization of *Toxoplasma gondii* from cats in Yunnan province, southwestern China. Parasit Vectors.

[19] Ge W, Sun H, Wang Z, Xu P, Wang W, Mu G (2014). Prevalence and genotype of *Toxoplasma gondii* infection in cattle from Jilin province, northeastern China. Vector Borne Zoonotic Dis.

[20] Ong YC, Reese ML, Boothroyd JC (2010). *Toxoplasma* rhoptry protein 16 (ROP16) subverts host function by direct tyrosine phosphorylation of STAT6. J Biol Chem.

[21] Alvarez C, De-la-Torre A, Vargas M, Herrera C, Uribe-Huertas LD, Lora F (2015). Striking divergence in Toxoplasma ROP16 nucleotide sequences from human and meat samples. J Infect Dis.

[22] Cheng W, Liu F, Li M, Hu X, Chen H, Pappoe F (2015). Variation detection based onnext-generation sequencing of type Chinese 1 strains of *Toxoplasma gondii* with different virulence from China. BMC Genomics.

[23] Reese ML, Zeiner GM, Saeij JPJ, Boothroyd JC, Boyle JP. Polymorphic family of injected pseudokinases is paramount in *Toxoplasma* virulence. Proc Natl Acad Sci USA. 2011;108(23):9625–30. 10.1073/pnas.1015980108 PMC3111280 21436047

[24] Saeij JP, Boyle JP, Coller S, Taylor S, Sibley LD, Brooke-Powell ET (2006). Polymorphic secreted kinases are key virulence factors in toxoplasmosis. Science.

[25] Hunter CA, Sibley LD (2012). Modulation of innate immunity by *Toxoplasma gondii* virulence effectors. Nat Rev Microbiol.

[26] Behnke MS, Khan A, Lauron EJ, Jimah JR, Wang Q, Tolia NH (2015). Rhoptry proteins ROP5 and ROP18 are major murine virulence factors in genetically divergent south American strains of *Toxoplasma gondii*. PLoS Genet.

[27] Ossorio PN, Dubremetz JF, Joiner KAA (1994). Soluble secretory protein of the intracellular parasite *Toxoplasma gondii* associates with the parasitophorous vacuole membrane through hydrophobic interactions. J Biol Chem.

[28] Craver MP, Knoll LJ (2007). Increased efficiency of homologous recombination in *Toxoplasma gondii* dense granule protein 3 demonstrates that GRA3 is not necessary in cell culture but does contribute to virulence. Mol Biochem Parasitol.

[29] Dubey JP, Van Why K, Verma SK, Choudhary S, Kwok OC, Khan A, et al. Genotyping *Toxoplasma gondii *from wildlife in Pennsylvania and identification of natural recombinants virulent to mice. Vet Parasitol. 2014;200(1–2):74–84. 10.1016/j.vetpar.2013.11.001 PMC4526132 24332401

[30] Shwab EK, Jiang T, Pena HF, Gennari SM, Dubey JP, Su C (2016). The ROP18 and ROP5 gene allele types are highly predictive of virulence in mice across globally distributed strains of *Toxoplasma gondii*. Int J Parasitol.

[31] Shwab EK, Zhu XQ, Majumdar D, Pena HFJ, Gennari SM, Dubey JP (2014). Geographical patterns of *Toxoplasma gondii* genetic diversity revealed by multilocus PCR-RFLP genotyping. Parasitology.

[32] Etheridge RD, Alaganan A, Tang K, Lou HJ, Turk BE, Sibley LD (2014). The *Toxoplasma* pseudokinase ROP5 forms complexes with ROP18 and ROP17 kinases that synergize to control acute virulence in mice. Cell Host Microbe.

[33] Rosowski EE, Lu D, Julien L, Rodda L, Gaiser RA, Jensen KD (2011). Strain-specific activation of the NF-kappaB pathway by GRA15, a novel *Toxoplasma gondii* dense granule protein. J Exp Med.

[34] Saeij JP, Coller S, Boyle JP, Jerome ME, White MW, Boothroyd JC (2007). *Toxoplasma* co-opts host gene expression by injection of a polymorphic kinase homologue. Nature.

[35] Jensen KD, Wang Y, Wojno ED, Shastri AJ, Hu K, Cornel L (2011). *Toxoplasma* polymorphic effectors determine macrophage polarization and intestinal inflammation. Cell Host Microbe.

[36] Yamamoto M, Standley DM, Takashima S, Saiga H, Okuyama M, Kayama H (2009). A single polymorphic amino acid on *Toxoplasma gondii* kinase ROP16 determines the direct and strain-specific activation of Stat3. J Exp Med.

[37] Henriquez FL, Nickdel MB, McLeod R, Lyons RE, Lyons K, Dubremetz JF, et al. *Toxoplasma gondii* dense granule protein 3 (GRA3) is a type I transmembrane protein that possesses a cytoplasmic dilysine (KKXX) endoplasmic reticulum (ER) retrieval motif. Parasitology. 2005;131(Pt 2):169–79. 10.1017/s0031182005007559 16149193

[38] Kim JY, Ahn HJ, Ryu KJ, Nam HW (2008). Interaction between parasitophorous vacuolar membrane-associated GRA3 and calcium modulating ligand of host cell endoplasmic reticulum in the parasitism of *Toxoplasma gondii*. Korean J Parasitol.

[39] Shen B, Brown KM, Lee TD, Sibley LD (2014). Efficient gene disruption in diverse strains of *Toxoplasma gondii* using CRISPR/CAS9. MBio.

[40] Khan A, Fux B, Su C, Dubey JP, Darde ML, Ajioka JW, et al. Recent transcontinental sweep of *Toxoplasma gondii* driven by a single monomorphic chromosome. Proc Natl Acad Sci USA. 2007;104(37):14872–7. 10.1073/pnas.0702356104 PMC1965483 17804804

