# Supplementary figures and images for: Genotyping of polymorphic effectors of Toxoplasma gondii isolates from China
Source: Parasit Vectors. 2017 Nov 21;10:580. doi: 10.1186/s13071-017-2527-4 (PMC5697216; doi:10.1186/s13071-017-2527-4)

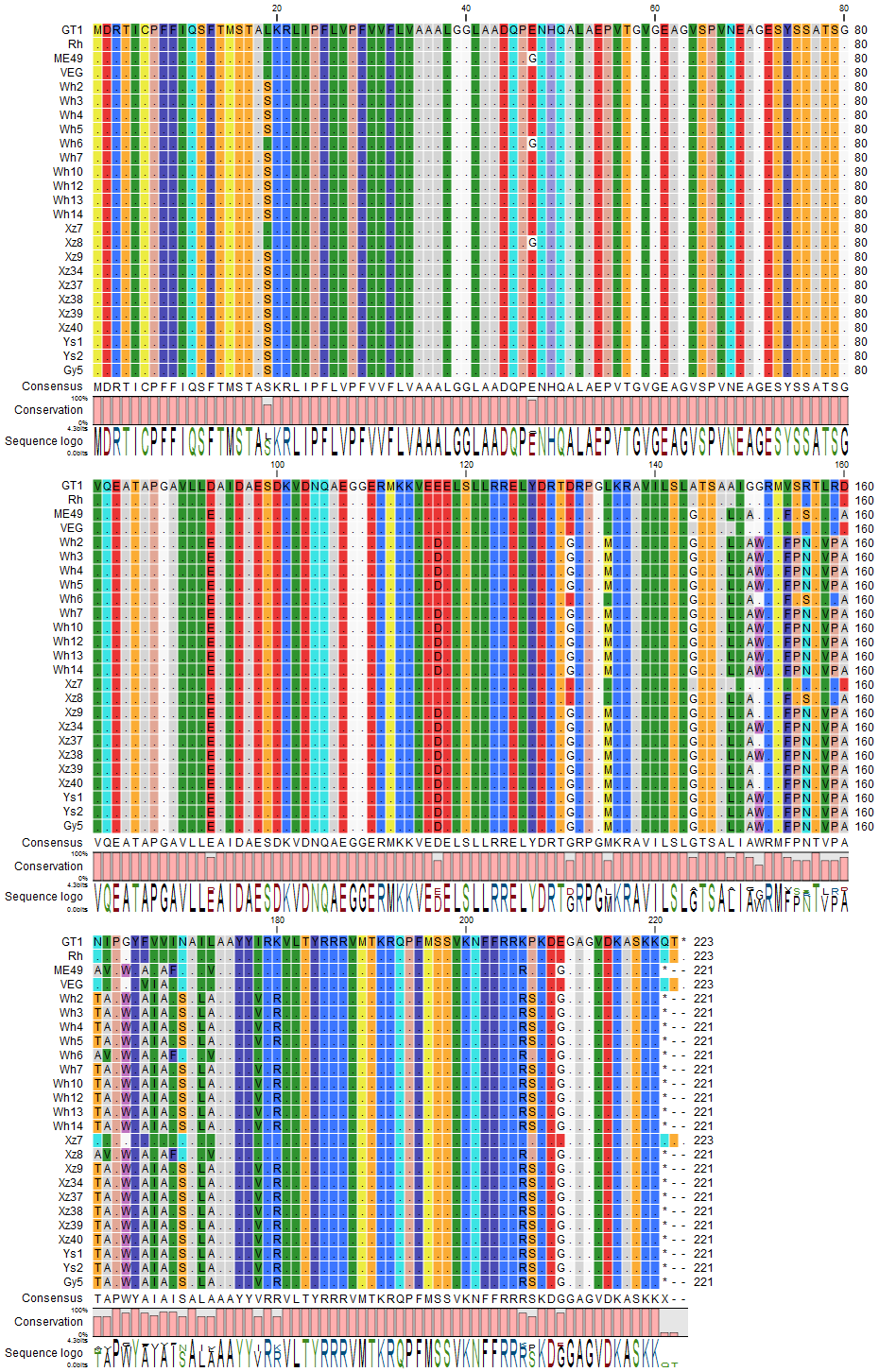

Supplement: Supplementary file 1 — GRA15 translations alignment. Analyzing the full length of GRA15, the result shows that all Chinese 1 and ToxoDB #205 strains (but not Xz7 strain) were identical to the type II strain, which has an 84 aa deletion from position 519 to 602. (TIFF 3957 kb) [file 13071_2017_2527_MOESM1_ESM.tif]

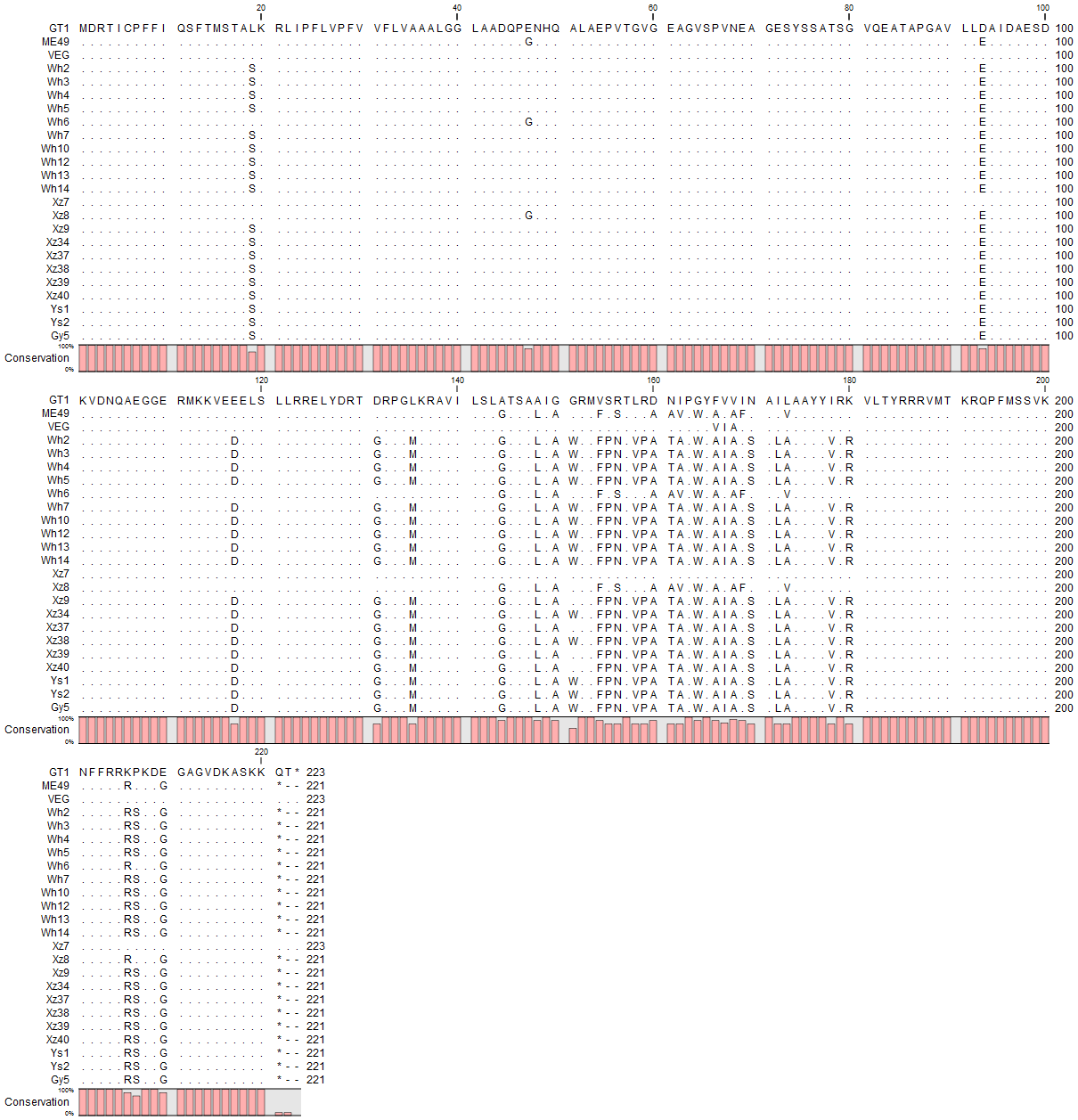

Supplement: Supplementary file 2 — GRA3 translations alignment. Aligning the full length of GRA3 translations of those strains, shows that Xz7 is the only one which has a longer length compared with the others. GRA3 of Wh6 and Xz8 strains are homologous to that of type II. The amino acid sequence from 131 to 180 is the mutation cluster region constituting a major part of all mutations. (TIFF 4457 kb) [file 13071_2017_2527_MOESM2_ESM.tif]
